# Editorial: Emotion dynamics: mapping the structure and function of emotional variability

**DOI:** 10.3389/fnhum.2025.1603774

**Published:** 2025-04-15

**Authors:** Jared J. McGinley, Derek P. Spangler

**Affiliations:** ^1^Department of Psychology, Towson University, Towson, MD, United States; ^2^Department of Biobehavioral Health, the Pennsylvania State University, University Park, PA, United States

**Keywords:** affect, emotion dynamics, emotion, emotion variability, temporal variability

Emotion has long proven to be challenging to study. This is because emotions are often ephemeral and amorphous, involving multiple response systems that vary dynamically in their expression over different timescales and individuals. The unfolding of emotion regularly interacts with regulatory processes and is shaped by complex variation in both intrapersonal processes (e.g., changing goal states) and environmental interactions with persons and objects. These dynamic features have resulted in definitional, measurement, and analytic challenges for the scientific investigation of emotion.

Despite its importance, *emotion dynamics* as a formal topic of scientific interest emerged only recently (in the last several decades), drawing attention from many disciplines including psychology, neuroscience, and computer science. This interest stems from the increased recognition that emotion is a complex time-varying and multivariate process (Kuppens and Verduyn, 2017) as well as the improvements that dynamics have provided beyond mean measures of emotion for predicting important outcomes such as symptoms of psychopathology (e.g., Sperry et al., 2020; Trull et al., 2015) and relationship outcomes (e.g., Butler, 2011).

This Research Topic was engendered with the goal of advancing the collective understanding of the dynamic nature of emotion through spotlighting different conceptual approaches, measurement tools, and quantitative techniques used for studying this phenomenon. We have previously expended our own efforts in service of similar goals. We have tried to index the multivariate (McGinley and Friedman, 2017) and temporally dynamic (Spangler and McGinley, 2020) elements of emotion; to improve quantification approaches for its measurement (Brouwer et al., 2018); to map out its nonlinear relationships with relevant outcome variables (Spangler et al., 2015); and contributed to broad-scale initiatives to consolidate and elucidate the field's knowledge of affective processes (Pace-Schott et al., 2019; Schiller et al., 2024). Accepting our own limitations in knowledge and expertise, we have embraced the wisdom of the familiar idiom, “it takes a village to raise a child”. In response, we invited the village of emotion scientists to foster our understanding of emotion dynamics.

From our perspective, the literature of emotion dynamics can benefit from areas of refined inquiry and improved methodology. Notably, we have approached this Research Topic with the view that there are needs to (1) better characterize the temporal structure of emotion dynamics and show systematic relationships between emotion dynamics' measures and established forces that influence and modulate their expression. In other words, if temporal dynamics in emotion reflect a real phenomenon (as opposed to noise), then their structure should be somewhat similar across persons, and the variance in emotion dynamics should be explained by factors that theoretically modify emotion, and (2) if real and noteworthy, then temporal dynamics in emotion should also explain meaningful behavioral outcomes. That is, the criterion validity of emotion dynamics measures needs to be further established. In this Research Topic, several empirical studies have contributed to addressing these needs.

## What is the temporal structure of emotional dynamics and what forces can be shown to modulate those dynamics?

Seah and Friedman affirm findings from existing literature that affective processes unfold quickly and can be indexed by both somatic and autonomic indicators. Through the use of cardiac and facial muscle indicators, they demonstrate that responses to emotional stimuli can dynamically change over the course of short time periods. Their findings also provide clear evidence that the dynamic unfolding of these physiological indicators of emotion manifest from self-environment interactions, and that the magnitude and timing of the physiological responses can be modulated by culture. The expression of emotion is often inextricably linked to concurrent regulatory processes which shape it. Using directed tasks, Kreibig and Gross showed that the regulatory processes that shape the dynamic expression of emotion play out within seconds and can be captured through measures of facial muscle activation. Further, they demonstrate that different regulatory processes have discernable time courses which can be indexed by separate indicators within a single response system (i.e., different facial muscles). Combined, these studies demonstrate that intrapersonal processes (coping) and extrapersonal influences (culture) can shape the time course and magnitude of affective processes of multiple response systems.

## Do emotion dynamics predict behavioral outcomes?

In a single-day multi-session competitive computer task paradigm involving on-the-fly learning and measures of attentional performance, North et al. used mean and variability measures of emotion to predict gaming performance. Participants were financially incentivized to perform well in 14 gaming sessions. Emotion metrics were derived from calculations of inter-session self-report of emotion responses. Of all the variability measures, flux, was the only out of many to emerge as a meaningful predictor of performance. In line with past research, they provide evidence that “shiny” new metrics of emotional variability are not always improvements over standard mean-based measures of emotion in predicting outcomes of interest. However, Johal and Ferrer advanced beyond traditional mean-focused measures of emotional interaction in romantic relationships. Specifically, they leveraged vector autoregressive modeling to quantify temporal change in dyadic emotional interactions. Such temporal change in the couples' emotional interactions explained unique variance in their relationship outcomes, over and above mean-based metrics. Collectively, these studies demonstrate that measures of dynamics can be useful in predicting behavioral outcomes, but that all measures are not made equal, and modeling approaches need to be thoughtfully applied to the research question of interest.

Beyond the specific themes above, other important theoretical and methodological points emerged from the articles in this Research Topic. Notably, metrics that capture emotion dynamics are inherently tethered to the theoretical models from which they're born—e.g., self-report metrics derived from the four quadrants of the Circumplex Model instead of more non-linear, multidimensional, or discrete models of emotion, as seen in North et al.

As indexed by the tight temporal coupling of emotion expression seen in Seah and Friedman with the regulatory processes indexed by Kreibig and Gross, a door is opened to improve our understanding of the influences that shape the unfolding of emotional expression. A Jamesian' perspective of emotion (see Friedman, 2010) would view the physiological expression of emotion as being produced before being tagged by the brain. Kreibig and Gross demonstrate that regulatory processes (presumably reflecting brain-based control or “tagging”) come online early in ways that potentially change the subsequent physiological response, thus morphing the initial “Jamesian” physiological reflexes in a complex manner. It is also likely that appraisals binned into primary and secondary processes (Lazarus and Folkman, 1984) are much too simplistic, and that cognitive processes are continually shaping the dynamic response of emotion expression though a constant feedback loop between the body and the brain which changes the goals and/or neural programs that drive emotional expression at any given moment. Future studies should look more closely at the activity of the brain and the body at the same time to improve understanding of the brain's dynamic processes in shaping the body-based emotion responses. Lastly, many studies of emotion dynamics use short-term or within-session calculations of dynamics. As shown in Seah and Friedman and Kreibig and Gross, these can be useful for modeling the real-time unfolding of emotion processes. When predicting downstream consequences, however, events in the distant future (i.e., 1–2 years later) might be more usefully informed by dynamics' measures calculated over days or months (e.g., Johal and Ferrer).

Finally, we are grateful to the authors for their contributions to our collective knowledge of emotion dynamics, as well as to the reviewers who dedicated their time and expertise to shape these manuscripts into the valuable scholarly works featured in this Research Topic.
